# The collapse of the wave function as the mediator of free will in prime neurons

**DOI:** 10.3389/fnins.2025.1637217

**Published:** 2025-08-21

**Authors:** Diego A. Loboguerrero

**Affiliations:** Cell Signaling Laboratory, Institute of Experimental Medicine, Universidad Central de Venezuela, Caracas, Venezuela

**Keywords:** collapse of the wave function, free will, soul/spirit particle, quantum biology, consciousness, spontaneous activation of neurons, prime neurons

## Abstract

In our current view of reality, free will hangs on two opposing forces. On one side, we have determinism, which states that everything is already determined by our inner constituents, the atoms and molecules that form our bodies. On the other side, we have quantum mechanics and its view that everything in the quantum world is inherently random and probabilistic. None of these perspectives gives rise to the phenomenon of what we call free will, but here in this article, we provide an underlying mechanism for how free will should operate in our world. We propose that the collapse of the wave function is responsible for determining our free will in prime neurons. The collapse of the wave function is the process by which a particle passes from a state of superposition or being in several places at the same time to a definitive state with clearly established properties. Prime neurons are a class of neurons that are responsible for initiating a thought process or an action in our brains. But for this to operate, the collapse of the wave function must not act on regular matter, for that would yield a purely random result. We need a new and hypothetical particle, for which we have placed the term “soul/spirit particle.” This soul/spirit particle has very specific features, as we have discussed in this article, and we have provided a mathematical model to explain its interaction with our inner physiology.

## 1 Introduction

We all have a strong inner conviction that we can make decisions and actions according to our own reflections, beliefs, preferences, and feelings. We assume that our choices were made in our consciousness and that we are held responsible for the decisions we make in our everyday lives. But that is not the view held by science, ever since the thoughts of Pierre-Simon Laplace, who stated that if we know to infinite precision all the variables around every particle in the Universe, we could see the future as well as the past (Guggisberg and Mottaz, 2013; Kožnjak, 2015). However, according to this view, free will should not exist because everything would have been predetermined by our own inner constituents, the atoms and molecules that form our bodies. This is what we call determinism, which is rooted in cause-and-effect relationships of the whole universe.

Nonetheless, along came quantum mechanics that challenged this perspective; at the heart of every process of electrons and subatomic particles is not deterministic, but rather probabilistic, as defined by the wave function of the particle. But it goes even beyond this scope, at the core of this interaction, the particle lies undefined or in a superposition of states, being in several positions at the same time until an observer “watches it” or when it interacts with the environment. Then the collapse of the wave function (CWF) happens, and the particle appears at a point position with clearly established properties in a purely random way according to the wave function of that particle (Jedlicka, 2017). In this oversight, the past can be reconstructed from the patterns that are defined after the CWF, but not the future, as it lies forever locked in quantum uncertainty until the present is made at the moment of collapse.

Nevertheless, this view does not save free will after all, because if the future is not defined and is rather probabilistic, does this mean that our free will is also random and probabilistic (Mudrik et al., 2022)? There is this notion of emergence that says that we cannot draw conclusions about our external world by the inner workings of its constituents, just like water is composed of H_2_O and wetness are two different things. We cannot say that our free will is random and probabilistic if we actually do not feel that our decisions are probabilistic and random (Joober and Karama, 2021; Mitchell, 2018). However, in the case of emergence, I believe we can know or understand the inner workings of our reality just by applying the science beyond a distinct conjecture, for example, why gold is that color or why mercury is a liquid at room temperature. Just by applying what we know of chemistry, we can understand that the relativistic effect on the outermost shell of mercury makes it a liquid because it rarely shares these electrons with other mercury atoms. And we know that gold is that color by the wavelength of light that the atom absorbs and reflects in its outermost shell of electrons. By knowing this and the root that explains many different things, we can conclude that emergence is wrong and that a deep understanding mechanism must explain what we perceive as free will; we cannot blame our shared knowledge for emergence just because we do not understand the root mechanism that explains a distinct conjecture (Schwartz et al., 2005).

Free will can be defined under three distinct conditions. First, there is the “ability to do otherwise,” which means that there is an option to choose from; if there is but one option, we would not be free to do what we please. The second condition is “control over one’s choices”; the subject that acts must be the same as the author of that choice, without interference from people and mechanisms outside of one’s reach. For example, a tic in the eye, an involuntary movement of the eyelid, even though the movement was made by the same subject by itself, does not count as free will, because the subject was not in control of that movement. The third condition is that the decision must be “responsive to reasons” according to the inner beliefs of that person. If our choices were random, then the subject would not be in control of his or her life, and everything would be based on random factors. No, decisions must be rationally motivated; if I marry based on the toss of a dice it is not me who is free to make a choice, even though I am free to say “I do,” but if I marry a woman by their ideas and by my deep love for her, then my decision is truly free (Lavazza, 2016).

But this is not how science post free will lately; ever since the experiments carried out by Libet et al. (1983) and others that came after him (Neafsey, 2021), they began to use EEG tracings to measure the time of volition, or the time we become conscious of our decisions. They instructed volunteers to watch a screen with a dot rotating around a clock face, and after the dot had gone at least once around the clock, they told the participants to make a voluntary movement whenever they wished. They were asked not to plan the movement ahead of time but to act spontaneously. Then they were told to report the time when they were aware of the “urge,” “intention,” or “decision” to move by stating where the dot was in the clock that was in front of them. They found that the brain already orchestrates the decision several milliseconds before they become conscious of their choices (Rigoni, 2010). They labeled this EEG tracing as the readiness potential (Triggiani et al., 2023) and argued that free will is just an illusion. Others state that the brain prepares itself for the decision to be made or that there is a veto power for every decision that will be carried out, the famous “free will not” or “free veto” (Liljenström, 2021). However, subsequent studies have shown that a readiness potential also precedes such conscious veto decisions before we sense the urge to move, making such an argument not defensible (Schurger et al., 2021). Yet, after repeating the experiment several times and in different ways, the conclusion is still the same; it appears that the subconscious mind decides before we become conscious of it.

However, along came Hameroff (2012), who challenged this idea by arguing for a quantum effect in this phenomenon. He stated that quantum objects can relay information backward in time. He based his assumption on a thought experiment carried out by Wheeler in 1978, where the choice of a particle entering one of the slits in the double-slit experiment was delayed until the particle or wave had passed over one or both of the slits at the same time. This famous double-slit experiment explains the dual nature of particles that act as waves and as particles simultaneously. When not observed or measured, the particle acts as a wave by giving the classical interference pattern at the other side of the slits; this is true even if we shoot one particle at a time. But when we place a detector to see which of the slits the particle went through, we notice that the interference pattern disappears and a particle distribution is seen on the other side of the slits, even if the particle went through the slit without the detector. In the case of Wheeler, which was confirmed later by Kim et al. (2000), the path of the particles was erased and delayed by distance to see an effect backward in time.

In another experiment that went further, Peres (2000) and Ma et al. (2012) used entangled particles in their delayed choice setup. Entanglement is a property where the state of one particle is correlated with another particle, no matter the distance they are apart. This is what Albert Einstein quoted as “spooky actions at a distance.” If two particles are entangled, if you measure the spin of a particle to be spin up, the other particle must be spin down, at the exact time that the first particle was measured, no matter if the distance between the two is millions of miles away, or even Galaxies apart. This bizarre effect has been documented and proven correct in countless experiments. In their setup, two pairs of entangled particles are separated, and one from each pair is sent to two measuring devices, each associated with a conscious observer, conventionally called “Bob” and “Alice.” A third observer, named “Victor,” decides to measure the two particles as an entangled pair or as separate particles, and this determines if Bob and Alice observe them entangled, showing quantum correlations, or as individual particles, with no entanglement between them. The strange effect was seen when Victor could decide whether Alice and Bob saw them as entangled particles or not after Alice’s and Bob’s devices had measured them (but before Alice and Bob consciously viewed the results).

Another possible explanation for Libet’s experiments and subsequent studies is the Two-State Vector Formalism (TSVF). In standard quantum mechanics, we typically describe a system using a single wave function, which evolves forward in time from the past. However, in TSVF, we require two wave functions: one evolving from the past to the future and another from the future to the past (Elitzur and Cohen, 2020). TSVF treats time symmetrically, like a river that flows both forward and backward. In this scenario, a “negative mass particle” affects an outcome in the past (Elitzur et al., 2018). So, in Libet’s experiment, the readiness potential stands for the past building up a neural activity, but the final outcome, whether the person moves or not, depends on a future measurement: the actual decision to act or not.

These tests show that in quantum mechanics, time is an elusive concept; what has been decided in the future could affect a state in the past. Therefore, what Libet and their colleagues have seen in their experiments may be the first glimpse that a quantum process is operating in our brains. For free will to be true, and we feel it to be genuine, a quantum effect must occur inside our skulls. Future experiments must be conducted to detect and discern if this statement is true. It is known that the brain is too wet and noisy for quantum effects to be prominent, but our latest research has detected quantum effects even in the harsh conditions inside our brains (Simon, 2018; Willeford, 2023). But what is to ponder is, what are these quantum effects operating inside our minds? And how do they correlate with our perception of free will?

Others have tried to locate a region in the brain responsible for free will, and they have found that brain damage in the anterior cingulate cortex affects our volition, or our desire to act, causing what is known in the medical setting as akinetic mutism. Lesions located in the precuneus affect our sense of responsibility for our actions, or what is called agency, and this lesion causes alien limb syndrome (Darby et al., 2018). However, other researchers suggest that free will resides in the supplementary motor area (SMA), which precedes not only certain simple motor actions but also the point at which we become aware of our intention to perform such actions. They claim that neurosurgical resection of the SMA gives akinetic mutism as well, but for a definite time. After 11 days or up to 3 months, the subjects recovered their sense of volition, with only remaining difficulties in performing rapidly, alternating movements of the hands (Sjöberg, 2021). Could this be evidence that there is no brain location for free will? Could this recovery point out that there is an underlying mechanism explaining free will? Could this fact point to a quantum phenomenon for free will?

## 2 Free will and the collapse of the wave function

Either way, how can we humans have free will while avoiding determinism of our inner constituents and at the same time prevent falling into the random chaos of quantum systems? If Libet’s experiments could be a hint to a quantum process in the brain, what would this mechanism be? There is a theory that explains consciousness that invokes quantum processes like the famous Orchestrated Reduction theory of Roger Penrose and Stuart Hameroff which invokes the cytoskeleton, more precisely, the microtubules, oscillating between two or more quantum states, and the combined computations of these in all neurons, just like a quantum computer, will give rise to what we know of as consciousness (Hameroff, 2022). However, can we propose a different theory that may explain free will and consciousness in a quantum framework?

One way to avoid determinism and randomness in explaining free will is to allocate the CWF as the source of that free will. The CWF is purely random and stochastic, but the CWF portending free will must not be random, but rather upon a person’s will. This CWF happens inside our brains, altering our inner physiology, not influencing our external world, as experiments of the double-slit experiments prove that we do not have external causation of our conscious mind (Walleczek and von Stillfried, 2020a,b; Radin et al., 2020). If we perform the double-slit experiment and we humans concentrate our minds to produce a given outcome, we cause nothing, even though the role of consciousness is not defined in what is called the measurement problem. In the measurement problem, we do not know where the CWF happens in reality. Does it happen at the detector, or does it happen when a measurement is recorded in its digital memory, or does it happen when a human eye observes it, or does it happen when our brains interpret the results? Does measurement require consciousness? (Sokolovski, 2020).

I am a strong advocate of the Copenhagen interpretation of quantum mechanics, which states that when a particle is in a superposition of states, it is both a wave and a particle at the same time, it both spin up and spin down at the same time, and it is a multiple places also at the same time. When the CWF happens, all its superpositions disappear, and a distinct particle with precise properties is detected. Other interpretations of this phenomenon fall short of their premises, like the many-world interpretation of quantum mechanics that argues that a completely new world branches on every reality of the wave function. I oppose this view because it ignores a different set of laws that also govern our reality, which is justice for every evil and wrongdoing that I may do. If all possible states of a quantum system happen, I am a rock star in one Universe, or I win the Nobel prize, or I die young. Still, there is a branch of the Universe where everything happens favorably, and nothing wrong is seen in my reality, which opposes the view of justice for every sin and wrongdoing I may do; God will not allow every possible outcome to become true and a reality.

There are other contenders to explain quantum phenomena, like Pilot-Wave Theory, which states that there is a guidance wave determining the path of the particle (Norsen, 2018). These and other theories attempt to overcome problems such as the measurement problem, non-locality, and indeterminism. Nevertheless, the current approach assumes the Copenhagen interpretation of quantum mechanics, and if found to be true, will make the Copenhagen interpretation stand out from the rest of the theories out there.

But what is the objective of this CWF? The target may be voltage-gated channels. We know that there are states in a voltage-gated channel; it can be in an open, closed, or inactive state (Palmer and O’Shea, 2015). These states can be in a superposition of states. When a choice happens by the will of a person, this channel may open or close based on the role of that channel or that neuron in defining our will. They exist in a superposition of states before settling into a classical state by the CWF. We just have to determine if these voltage-gated channels are in a superposition of states, and there are ways to do that. If the microtubules can be in superposition (Babcock, 2024), and they are composed of hundreds of amino acids, what makes us think that the voltage-gated channels cannot also be in a superposition of quantum states?

Other mechanisms may involve the CWF altering the state of the lipid bilayer, favoring the opening or closing states of gated channels inside the membrane of a neuron. These effects indirectly change the permeability of a channel by the state of the lipid bilayer (Cocchi et al., 2017). Another mechanism may be changing the electron chain inside a voltage-gated channel, modifying its permeability to the ions entering or leaving the neuron. Ion channels often have a selectivity filter that discriminates between many ions. The ions in this filter may exist in quantum superposition states before selecting a path through the hole of the channel. This will directly modify the permeability of a given channel to a certain ion. We can also have the CWF affecting the sensitivity of a ligand, a neurotransmitter, with its appropriate channel, increasing or decreasing its affinity, which in turn would modify the firing rate of that neuron. Lastly, the CWF may directly affect the ions near voltage-gated channels, activating them by managing the concentration near them. The end conclusion is the same in all these cases: the CWF will activate or deactivate the propagation of an action potential in a given neuron.

If I had to promote a mechanism, the CWF should directly alter the voltage-gated channels, opening or closing the channels based on our will. Still, more research needs to be done in this area to define “what in reality is in superposition (Khrennikov et al., 2018)?” And “what does the CWF do to trigger the action of a given neuron?” Proteins, including ion channels, operate in a realm where quantum effects may be prominent, especially in the transition between states. This makes them a natural target for wave function collapse. Protein folding and conformational dynamics are already being explored in quantum biology (Jedlicka, 2017), and future research may shed light on novel mechanisms that control neuron behavior.

## 3 Prime neurons

Now, the question may arise: what neurons behave like that? Which neurons are affected by the CWF? Are all neurons behaving this way? Well, not necessarily. I hypothesize that there is a class of neurons that behaves very differently from the rest. These neurons are the ones that initiate a thought process or initiate a given action in our brains, like the movement of an arm or of our hand. I call these neurons Prime Neurons (PN), and they are the ones more linked with the CWF. At first glance, these PN would be detected as neurons that fire spontaneously, without an apparent trigger from a neighboring neuron (Caspar and Cleeremans, 2015).

**Figure 1:**
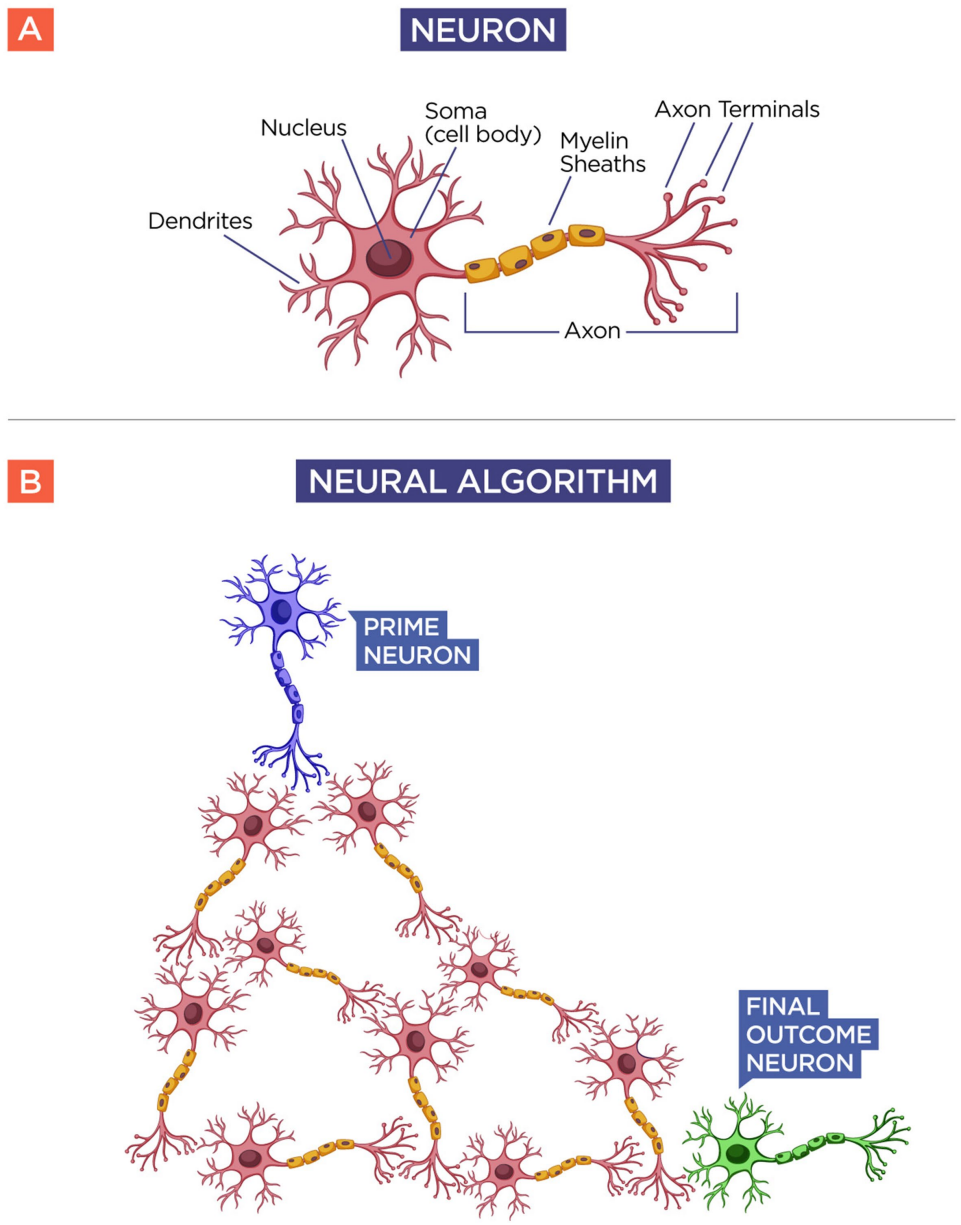
Neural algorithm. Panel **(A)** shows the schematics of a regular neuron. Notice that the neuron receives an input (dendrites) from other neurons and has an output mechanism (axon) that relays the action potential to the next neuron. If this is true, then all neurons must have an input and an output mechanism, but there should be one neuron that initiates the relay of information among all neurons. Panel **(B)** shows the schematics of a neural algorithm. By following the same idea, there should be a neuron or group of neurons that initiates a given stimulus called the “Prime Neuron” (Blue) for a given thought process or action taken by the “Final Outcome Neuron” (Green) after the information has been processed by the neural algorithm (Red).

To understand their existence and their implication, consider a simple model of a neuron (see Figure 1). A neuron is composed of a body with its nucleus and its dendrites, and an axon that springs from it, which is responsible for relaying the information, or the action potential, to its neighboring neuron. A group of neurons forms what I call a neural algorithm, basically a thought process in the brain. When you eat a meal, you usually eat every food in a related order, and have not you wondered why? When you eat a meal, you usually use a neural algorithm; you eat each food in an established order. I have witnessed people eating a single item to completion before moving on to the next on their plate. Then, in this example, the algorithm goes like this: “eat a single item until completeness before moving to the next.” On another example, I have witnessed people selecting a bite of each item based on, presumably, the preference of the moment. In this case, the neural algorithm would go like this: “Eat each bite according to your preference at that moment.” This is what a neural algorithm is. Yet if the vision of the neural algorithm is correct, we could have a lot of neurons relaying their information to other neurons in an endless chain, to a final outcome in a given action, but who started this neural algorithm? What neuron was responsible for initiating a thought process? We could envision that no neuron initiated the whole cascade of actions, that it all came from random fluctuations of multiple neurons connected in series. But even this is wrong because there must be a single neuron that initiated the whole neural algorithm.

Take the example of artificial intelligence, AI, like ChatGPT. ChatGPT could be very brilliant, having many algorithms to respond to any question presented to it. It can tell you what the weather could be tomorrow, or it could explain to you quantum mechanics in a very clever way. But by itself, it can do nothing, not even type a simple word. It needs a human to interact with it (Kastrup, 2017). This is an example of a PN. A PN is needed to start an interaction with the outside world. Surely, you could say that you could program ChatGPT to do things by itself, but you fall under the same premise; you need a human interaction to do that. By itself, ChatGPT can do nothing unless you program it to do so, but who programmed it to do things? You still need a human interaction to program ChatGPT. Then you could argue that I could program ChatGPT not only to have lone interactions but also to program itself, but you still fall under the same argument; you still need a human being to program ChatGPT to do all that. Another example to take away is the so-called “brain in a jar” (Kastrup, 2017). How much output would a human brain provide without sensory input? Is sensory input not a part of a chain reaction tied intrinsically to motor output? If PN exists, they should predict a motor output without sensory input.

PN behaves this way; they are needed to initiate an interaction with the environment. These are the neurons that make us human in a very specific way. If we did not have these neurons, we would be just a machine, having many neuronal algorithms and no interaction with the environment. In neuroscience, we have detected many neurons that act on themselves, that spontaneously fire without the need for previous stimulation (Dang-Vu et al., 2008; Lucas-Romero et al., 2024; Bukalo et al., 2013; Vyleta and Smith, 2011). Could these neurons correspond to PN? What is needed in research to detect these PNs? In a variant of Libet’s experiment, Schurger and his team (2012) detected spontaneous neural activity before the initiation of the readiness potential. Could this be a first hint of the existence of PN in the initiation of a movement? Could PN be initiating a decision and a movement before we become conscious of their interaction? Could PN be hidden under the schemes of these experiments (Schurger et al., 2012; Caspar and Cleeremans, 2015)?

## 4 Features of a hypothetical particle

Nevertheless, for the CWF to follow the will of a person, it cannot do it in the way it does with the rest of the particles of the entire Universe. These interactions between two particles of the regular world would be purely random as it is, as defined by the wave function of both of these particles, and we know that for free will to exist, it must not rely on random factors. Also, for a completely free will must be able to make a choice regardless of the surrounding environment. If the environment affects choices, then that will will not be free; it will be influenced by external factors. No, for the interplay to be in accordance with the will of a person, a hypothetical particle must be defined. This hypothetical particle at the CWF will closely follow the will of a person and not be completely random, as it is with the rest of the particles of normal matter. I call this hypothetical particle the soul/spirit particle (SSP), and it has very defined characteristics as we will discuss further along (Montague, 2008).

The first and main feature of this hypothetical particle is that this particle has the intrinsic ability to CWF according to “will” or intentionality, not like regular matter, which CWF probabilistically or through observation/interaction; this is central to our hypothesis. These interactions should not strictly follow the deterministic laws of classical physics. Instead, they would incorporate an element of choice or agency, differentiating from other quantum particles. This is what gives us the ability to decide without falling into determinism or the random factors of quantum mechanics; it is a unique ability that all humans share.

Secondly, it must be selective in its location. It should interact primarily (or exclusively) with the brain, particularly with neurons. At this point, we do not know if it acts on other cells apart from neurons, like astrocytes and oligodendrocytes, but we are left to speculate that they generally interact with PN, the neurons that initiate a thought process in the brain. As stated before, we do not believe that the CWF affects other regions apart from the brain, especially the regions of the brain where consciousness and decision-making take place, like the thalamus and other regions of thought integration (Whyte et al., 2024; Storm et al., 2024). This localization would explain how it influences free will and internal physiological processes without affecting external quantum systems. So, no interaction with the external world as we have seen with the double-slit experiments on a conscious outsider (Walleczek and von Stillfried, 2020a,b; Radin et al., 2020).

The SSP should exist in a coherent quantum state, and its interaction with regular matter in our brains must force the collapse of this regular matter to what dictates our will on these internal quantum systems. This coherent quantum state may make this particle elusive to be detected by our instruments, but it may imply that this particle may exist or have a presence in additional dimensions of reality, like those depicted in string theory or M theory (Başar and Güntekin, 2007). We still do not know where this hypothetical particle exists. Does it reside only in the presence of neurons inside our brain? Is it located in additional dimensions of our reality? Does it have mass? We are left to speculate and to ponder its existence until we have definitive tests on its reality.

The SSP should allow for a two-way interaction. First, from soul/spirit to brain, causing neuron activation or other physiological processes to facilitate free will, as we have discussed previously. Secondly, from the brain to the soul/spirit, potentially allowing feedback from the brain’s environment to the SSP, but we lack a mechanism for how this interaction should occur. However, in our numerous theories of consciousness, if our theory is proven correct, they should add this interaction of the SSP into account in their equations of consciousness (Mudrik et al., 2024).

To fit within the current frameworks of physics, the particle’s interaction should need to conserve energy and subtly comply with any other physical laws. This might involve influencing quantum probabilities rather than directly adding energy to the whole system. This will allow the study of these particles in our current framework of physics, if it is possible. The particle might have a distinct quantum signature or behavior, unlike any known particle, which could theoretically make it distinguishable if the right experiment were performed.

And what about features like charge, spin, and mass? Can we make any assumption about these features in our model of the SSP? If we consider the SSP as a bridge from the physical world to this new reality, fundamental properties like charge, spin, and mass should reflect its unique role. This insight could pave the way for treating it as a real physical entity rather than just a speculative idea. See Table 1 for a summary of these features.

**Table 1:** Hypothetical comparison to known particles.

Feature	Known particle comparison	Soul/spirit particle hypothesis
Charge	Neutral (Neutrinos)	Neutral or “hidden” charge in higher dimensions
Spin	Fermions (Spin ½)	Exotic or Higher-Dimensional Spin
Mass	Neutrinos (Extremely Low Mass)	Extremely Low Mass
Interaction	Weak Nuclear Force	Even weaker than the weak force, primarily quantum coupling

The first to consider is charge; the SSP should have a neutral or no electric charge. This envisions that the SSP should not interact with the electromagnetic field, for such interaction would cause an observable effect in our physical realm. If this were so, we would have detected its presence some time ago. This idea of the SSP having no charge would align with the concept that this hypothetical particle does not directly influence our external environment. It would create an interaction with neurons through the CWF but will not alter in any way our inner physiology, and even less our external environment. A neutral charge would help it remain “invisible” to traditional detectors that rely on charged particle behavior, like, for example, MRI or EEG (Jefferson Lab Qweak Collaborator, 2018).

If the SSP is quantum in nature, we propose it should have an exotic spin. If it could have a fractional spin like Fermions (like quarks and electrons), it would maintain individuality and avoid condensation into indistinguishable states, but we would be able to catch this SSP in a box, which we think would not be possible (Arndt et al., 2009). However, it may also have an exotic spin, like a higher-dimensional spin state or even a non-standard fractioned spin number. This may be because the particle may exist in an additional dimension apart from the three dimensions of space and one of time. It is unknown to us; we need to isolate this particle in order to determine its physical properties, but in the meantime, we are left to speculate about the properties of this particle with what we currently know of particle physics. We believe that condensation to a common state is not the appropriate behavior of the SSP, for it must maintain individuality to affect particular neurons, especially PN, but we believe that we cannot isolate this particle in a box like a regular electron would be; that is why we support an exotic spin state.

Concerning the properties of mass, this hypothetical particle must have extremely low or zero rest mass. We presume this based on the assumption that this particle may interact with regular matter of the brain without being detectable by any means. If it were too massive, we would have detected this influence in our experiments. This elusive particle may have a very low mass, but not zero, akin to neutrinos, which barely interact with matter but still have a measurable mass under certain conditions. It could also have a zero rest mass, which would allow it to move freely or exist “outside” of the spacetime realm. If this hypothetical particle exists in different dimensions of reality, it could have zero rest mass, but this could make the particle travel near or at the speed of light, which is why we speculate that it could have an extremely low mass under experimental conditions.

The interaction strength between the SSP with ordinary matter would have to be far weaker than the weak nuclear force, making it effectively unobservable through standard physical experiments. This could mirror how neutrinos interact, but even more elusive in a sense. We would not call it a force by itself, but rather a distinct particle with a strange influence over matter in the form of the CWF. This interaction would not give energy to the system in the same way as a force would because its interaction would solely be with the CWF with the particles of regular mass in voltage-gated channels.

Another property of this hypothetical particle could be that the SSP should have an extreme resistance to quantum decoherence. This would allow the particle to maintain its quantum properties indefinitely, which would support our theory that it could influence the brain states without succumbing to environmental noise. If this particle resides in another dimension of reality, like those expressed under string theory, we assume that quantum decoherence might be restrained from not sharing a common background with regular matter. This would not limit the CWF affecting ion channels where electrons and charges can exist in a quantum superposition of states, because the fact of being in a superposition also implies a superposition in all quantum dimensions, not only different positions in temporal–spatial grounds.

Finally, it may well be that this objective particle might carry mass or charge in hidden dimensions of reality, like in those theories that hypothesize additional dimensions of this world (String Theory or M-Theory), while appearing massless and neutral in the Observable Universe. This would allow it to exist in a hybrid state between these additional dimensions and our three dimensions of reality and one of time. I recognize that we are making a lot of assumptions here, but the world, including quantum mechanics, was made for it to be understood by the pieces presented to us by the realm of science.

## 5 A mathematical model

This section proposes a mathematical framework to describe the interaction between the non-physical soul/spirit and the physical brain, as hypothesized in the quantum free will theory. The model integrates principles of quantum mechanics and neuroscience, aiming to explain how conscious choices emerge from the interaction of the soul/spirit with neurons. Here, we build our model based on previous work that merges quantum mechanics with the field of neuroscience (Hameroff and Penrose, 2014), and we propose our original idea that departs from existing ideas in the field (Tegmark, 2000).

In order to build our model, we have used these key assumptions: First, the soul/spirit is distinct from physical matter particles but can influence wave function collapse. Neurons operate as quantum systems prior to firing, existing in a superposition of firing and non-firing states by the interaction of voltage-gated channels. The soul/spirit influences this superposition, collapsing the wave function according to the will of the person. The effects of this collapse are local to the brain and do not extend to external quantum systems.

Neurons exist in a superposition of two quantum states (Koch and Hepp, 2006), firing or non-firing states, either by affecting voltage-gated channels or by any other means as seen in Equation 1 (Arndt et al., 2009):


(1) ∣Ψneuron〉=α∣0〉+β∣1〉

Where:

∣0⟩: Neuron is inactive.

∣1⟩: Neuron is firing.

∣α∣2 and ∣β∣2 : Probabilities of each state.

These neurons exist in a superposition of states until the CWF defines a definite state upon interaction with the SSP (Khrennikov et al., 2018). These neurons exist in superposition, no matter the mechanism between the CWF and the state of the membrane’s ion channels, because what is important here is the generation of the action potential or not by the interaction with the SSP.

The soul particle influences the CWF through a soul-particle operator ${\bar{H}}_{\text{soul}}$ . This operator modifies the probability of neuron states based on the will of the soul (see Equation 2).


(2) $${\bar{H}}_{\text{soul}}=g\cdot {\bar{W}}_{\text{spirit}}$$

Where:

g is the coupling constant representing the strength of the soul-brain interaction.

${\bar{W}}_{\text{spirit}}$ represents the will of the soul/spirit and operates on the neuron’s wave function.

The probability of collapse to ∣1⟩ (Neuron firing) is adjusted as seen in Equations 3 and 4:


(3) $$p_{\text{collapse to}1}=|\beta |2+g\cdot \lambda$$

(4) $$p_{\text{collapse to}0}=|\alpha |2+g\cdot \lambda$$

Where λ quantifies the intentional influence of the soul/spirit on the neuron. The more influence of the SSP has on the neuron, the greater λ and more power it has over the firing of the neuron, which in this case would represent a PN. g in this case is a constant in nature and its value has to be determined in future experiments, but it is influenced by the strength of the SSP in affecting the CWF in PN (Stapp, 2001; Kauffman, 2016). A larger g means that the soul has more control.

The neuron state transition influences downstream neural activity. Using classical neural models like Hodgkin-Huxley or integrate-and-fire (Izhikevich, 2003), the firing probability of a neuron i could be described as (see Equation 5):


(5) $$P_{i}=f(V_{i},\lambda )$$

Where:

$V_{i}$ is the membrane potential of a neuron.

f is a function incorporating quantum collapse due to λ.

In the case of PN activation, let $N_{\left(t\right)}$ represent an activity state of a “Prime Neuron” over time, where $N_{\left(t\right)}=1$ denotes activation and $N_{\left(t\right)}=0$ denotes inactivity, therefore, the activation is governed by Equation 6:


(6) $$\frac{\mathrm{dN}(t)}{\mathrm{dt}}=g(\bar{w},E,t)$$

Where g is a function describing the interaction between the soul/spirit particle $\left(\bar{w}\right)$ , the local brain environment (E), and time (t).

Neurons do not work alone – they form neuronal networks. When one neuron fires, it can affect many others. The influence of just one neuron can propagate through the brain’s neural network. Therefore, this next model uses a standard neuroscience equation to show how neurons interact (see Equation 7) (Deco et al., 2010):


(7) $$\frac{{\mathrm{dV}}_{i}}{\mathrm{dt}}=-\frac{V_{i}}{\tau }+\sum_{J_{\mathrm{ij}}^{W}}f(V_{j})+{\bar{H}}_{\text{soul}}$$

Where:

$V_{i}$ stands for the membrane potential of neuron i.

τ stands for the time constant for neural recovery.

$W_{\mathrm{ij}}$ is the synaptic weight from neuron i to neuron j.

$f(V_{j})$ is the firing rate of neuron j.

${\bar{H}}_{\text{soul}}$ represents the influence of the soul/spirit.

The SSP not only affects one individual neuron but also acts on the PN. This would, in turn, influence complete neural circuits and neural networks. However, its influence can be discerned by the given equation by an operator outside of the neural network, as can be seen in Equation 7.

The interaction between the SSP with the brain should reduce local entropy by introducing order to the system given by the next Equation 8 (Tononi, 2004):


(8) $$S_{\Delta }=S_{\text{before}}-S_{\text{after}}$$

Where S is the Shannon entropy of the neural system (see Equation 9):


(9) $$\mathrm{\Delta S}=-\kappa_{b\sum_{i}P_{i}}\;\ln p_{i}$$

Where:

$p_{i}$ represents the probability of the i-th neuronal state (firing or non-firing).

$\kappa_{b}$ stands for the Boltzmann constant.

Reduction in entropy ΔS represents the system moving from a state of high-entropy (random) to one of lower-entropy (ordered) by the influence of the soul (Tarlacı and Pregnolato, 2016). This reduction of entropy reflects purposeful, non-random decisions made by the soul. This change in entropy can be measured, and it could be the basis for detecting the influence of the SSP in neuronal circuits, to be proposed by future studies of quantum biology in the brain (Jedlicka, 2017).

To explicitly model the collapse mechanism (von Neumann, 2018; Sokolovski, 2020), we establish a collapse operator as seen in Equation 10:


(10) $$\bar{C}=\lambda_{\text{soul}}\cdot {\bar{P}}_{\text{spirit}}+(1-\lambda_{\mathrm{sou}l})\cdot {\bar{p}}_{\text{physical}}$$

Where:

$\lambda_{\text{soul}}$ represents the strength of the soul’s influence.

${\bar{P}}_{\text{spirit}}$ stands for the projection operator representing the soul-driven collapse.

${\bar{P}}_{\text{physical}}$ is the projection operator representing physical-driven collapse, like, for example, environmental decoherence.

This operator balances the contributions of the soul and physical realm in the decision-making process (Wigner, 1995). This model explains that there may be an influence from the external environment in our decision-making process, but if the theory is correct, this influence of the external environment should be negligible.

We could expand the model to include quantum biological processes like microtubule coherence or voltage-gated ion channels. We could define a coherence function C(t) for quantum systems in the brain (see Equation 11) (Hameroff and Penrose, 2014):


(11) $$C(t)=\psi (t)|\overset{¯|}{H}\psi (t)$$

Where $\bar{H}$ is the Hamiltonian of the brain’s quantum system. If we assume the SSP modulates C(t) then this would lead to Equation 12:


(12) $$\frac{\mathrm{dC}(t)}{\mathrm{dt}}=\alpha \cdot W(t)-\beta \cdot D(t)$$

Where:

W(t) represents will-driven interaction.

D(t) represents the decoherence effects of the environment.

*α*, *β* are scaling constants.

With these equations in hand, we can build computer models to simulate the brain/soul interaction and how volition takes its place. What is more important is that this mathematical model opens up an avenue for future studies concerning the SSP and its interaction with brain matter. We know from past studies on free will led us to a roadblock in our understanding (Libet, 1999; Haggard, 2008), because we could not gain insight into why unconscious decisions preceded conscious volition. However, with this model, we can venture into a path that unites neuroscience and quantum mechanics, and if we do it right, this will enhance our understanding of how free will really works. What is important to understand is that this mathematical model makes predictions, and these predictions can be measured to test whether these assumptions are correct or not.

## 6 Road to prove this theory

Testing this hypothesis will be challenging, but it is not impossible. Many researchers may see this as a realm outside the scope of science, but as science advances, we could venture into unexplored areas of consciousness and what lies above it, which is the influence of the SSP on brain matter. To test this theory, we need to unite two unique fields: quantum mechanics, or the study of the small scales of matter, with neuroscience, or the life science behind our mind, brain, and consciousness. As these two fields advance, they overlap, creating major leaps and breakthroughs between the two that will help us understand what is happening inside our skulls (Tuszynski, 2020; Franco, 2022). Having this hypothesis and this mathematical model could open a road for new advances in both these fields as we strive to prove this theory either right, with all its implications, or not.

Our theory suggests that some neurons fire without prior activation from other neurons due to wave function collapse by the influence of the SSP; this assumption can be tested, first, by the use of fMRI or EEG to analyze neural activity and look for spontaneous neural activation that lacks preceding signals (Caspar and Cleeremans, 2015). We could use fMRI or EEG to monitor neural activity in real-time during deliberate, conscious action, and search for neurons that activate without prior physical triggers (no preceding synaptic input or external stimulus) (Schurger et al., 2012). Some neurons should display activation patterns that cannot be explained by classical chain-reaction neural signaling or stochastic quantum effects. As a control group, we could use neurons that fire randomly and that are not influenced by the SSP, like tonic neurons. These tonic neurons are in charge of maintaining awareness and ensuring that all neurons are in an active state (Apicella, 2002).

We could also use single-neuron recordings to detect neurons firing without a physical neuronal trigger (Liu et al., 2021). If such activity exists, it would suggest a non-deterministic root. We should use human models to detect these PN since we speculate that in our uniqueness we should host these types of neurons, but animal models should also be done because they could gather valuable insights on how these cells evolved from single-cell organisms to the complex organisms that we are today.

The SSP could sustain quantum coherent states in neurons, especially in PN, but in other neurons as well, allowing free will to emerge. We should investigate whether quantum coherence is maintained in brain structures like in microtubules, as proposed by the Orch-OR theory (Hameroff and Penrose, 2014). We know that networks of tryptophan, like those found in microtubules, display ultraviolet superradiance, meaning that they exist in a quantum superposition of states (Babcock, 2024). We could use this technology to determine if voltage-gated channels also display superradiance activity, which in turn would mean that they also display quantum coherence or exist in a quantum superposition of states. And we should go beyond this scope, we should search for unexplained coherence persistence on different aspects of the neuron that resist environmental decoherence, we have to define what structures in the brain are in quantum superposition that might yield a quantum effect on consciousness. Prolonged quantum coherence in brain regions (for example, during decision-making) could point to an external influence stabilizing these states.

The SSP interacts only with the brain’s quantum system, not external ones, as we have seen. However, we can conduct the quantum double-slit experiment in living biological tissues or in neurons to see if we detect a difference. If the SSP interacts selectively in the brain, we might observe a unique quantum behavior when neurons or microtubules are involved, but it must be done right. Brain-based quantum systems should exhibit deviations from the expected collapse patterns observed in external quantum systems when done correctly. We can certainly design experiments that would be analogous to the quantum double-slit experiment, but performed in the brain regions inside our living skulls. But, as stated before, with our minds we cannot influence external environments outside our brains (Walleczek and von Stillfried, 2020a,b; Radin et al., 2020).

If the SSP links the physical brain with this new realm, it might maintain entanglement with other particles and systems. We should attempt to recreate entanglement between brain structures and other external structures, like photons and electrons. We could measure whether the brain introduces unexplained or unique entanglement patterns that differ from standard quantum predictions. The brain should exhibit non-local correlations consistent with the influence of this hypothetical particle. Quantum effects like entanglement, quantum coherence, and quantum tunneling have been detected in other biological systems, like in photosynthesis and bird magnetoreception (Arndt et al., 2009). We could test whether the brain could harbor similar effects, or it could even have more subtle quantum interactions. If free will relies on wave function collapse, this could mean that quantum processes play a role in brain function. As the field of quantum biology advances, we will be able to detect these subtle influences in brain function.

The SSP must interact with brain matter without violating in any way energy conservation. We could very well measure energy flows and fluctuations during intentional, conscious actions. We could also use ultra-sensitive calorimetry to detect tiny, unexplained energy states within neurons. Conscious decisions might correlate with subtle energy shifts that cannot be accounted for by known current physical processes. Other physical properties of the SSP would be more difficult to determine; however, as technology advances, we will be able to characterize these features in more detail.

Conscious free will, driven by the SSP, might create detectable patterns in decision-making or in any reactions. We could set up tasks where participants must make choices under conditions designed to amplify potential subtle quantum effects, like, for example, time pressure, randomness, or high uncertainty. It could well be that the effect of Libet’s experiment might be an example of a quantum effect yet to be discovered (Guggisberg and Mottaz, 2013; Hameroff, 2012). We may also search in experiments for patterns suggesting a non-random collapse of quantum probabilities that aligns with intentionality. We could even use participants to make choices between quantum-randomized options, testing if conscious intent biases the outcome beyond what we know of chance. We could compare brain activity patterns of subjects before and after decision-making to see if there is a non-deterministic “gap” where the conscious will might act. It may well be that data might reveal decision-making patterns inconsistent with either purely probabilistic quantum behavior or non-stochastic deterministic patterns.

If the SSP interacts uniquely with the brain, it may exhibit different behaviors in altered states of consciousness. Conditions like near-death experiences, out-of-body experiences, and even more commonly, patients in a coma or altered state of consciousness, might express physiological signatures that can be found in EEG patterns or other neuron activity tests (like fMRI) that correlate with the interaction between the SSP and the brain matter (Mudrik et al., 2024; Schiff, 2024). We should analyze thoroughly to detect any unique quantum effects or anomalies during these altered consciousness states. Unusual quantum phenomena in the brain during altered consciousness could point to the presence of an SSP.

The SSP might influence organic brains, not artificial systems. We should compare decision-making and other quantum processes in human brains vs. artificial neural networks (Feather et al., 2023). We should look for quantum phenomena or free-will-like behaviors that are absent in these artificial systems. Organic systems might show unique quantum behaviors tied to the SSP that are impossible to replicate artificially. Experiments should be done to detect differences between artificial intelligence and the human brain, like the presence or absence of PN.

If the SSP resides in another set of dimensions, outside of the classical three dimensions of space and one of time, bridging our physical world with this new realm, it might operate outside our normal experience of time. Time may be the key that could point to a quantum behavior. What Libet and his colleagues have found about this time difference could mark the beginning of a quantum behavior in the brain (Hameroff, 2012). We should conduct experiments involving the perception of time during moments of conscious choice or meditation. We should investigate whether decision-making processes show any deviation from expected temporal patterns. Time-sensitive experiments might reveal many things indicative of this particle’s influence.

Finally, we have our mathematical model; with this model, we can build computational algorithms that may simulate brain activity in humans. We can also make predictions based on this model, and we can test and see if these predictions come to life. A particular point to test would be measuring entropy in the brain (Lynn et al., 2021; Saxe et al., 2018). We could devise experiments to see if any pattern of behavior might influence the total entropy inside the brain (Hull and Morton, 2023). The total entropy might reflect the existence of this hypothetical particle. Any deviation from the predictive entropy of the brain could point to the existence of this elusive particle (Ingo et al., 2014).

## 7 Conclusions and implications

Here we present to science an intriguing hypothesis. Some may argue that it does not correspond to the realm of science, but if we can bring to the table a mathematical model and ways to test it and prove this theory, then why should it be this way? We as humans wake up in a world where there are puzzles everywhere, with pieces to discover here and there. An incomplete puzzle would be unfair to us; we should be able to build a complete puzzle of everything with the pieces that are handed down to us. We may not have evidence of everything, but the pieces that we have in our hands could point to other pieces in the puzzle as we solve the problems of life. What happens after we die should not be a question outside of the realm of science. If we learn to understand what has been given to us, we may see the truth, even though we do not have all the scientific rigor for every question that we ask.

So it is with the question of consciousness; many have embarked on a quest to find what it is, but just the mere fact of defining the question is very challenging (Bond, 2023). I do not know what consciousness is or what it is composed of Noirhomme et al. (2010), but I do have a piece of this puzzle. I do not know why consciousness comes and goes as we lay to sleep (Tononi et al., 2024), or what happens in the altered state of consciousness (Schiff, 2024), or how an anesthetic works (Franks and Lieb, 1990; Vadakkan, 2015), but what I do know is that if we believe that free will is a real and tangible thing, then this knowledge will point out to the existence of a hypothetical particle that may lie in a realm outside the three dimensions of space and one of time.

What Libet et al. (1983) have discovered could very well be evidence of a quantum effect in the brain (Neafsey, 2021). This notion is better and healthier than throwing free will out the window. But how can we know? What I do know is that we begin this quest with an underlying theory as displayed in this project. PN could be the key to this insight. Without the soul, neurons would fire randomly, based only on probabilities, or they would fire deterministically, influenced by the external environment. With the soul, neuron firing becomes intentional and reflects the will of the soul/spirit. What other researchers have found about the spontaneous activation of some neurons could be the first glance at the existence of these new hypothetical neurons (Vincen-Brown et al., 2016).

But we should consider, for a while, the implications of this theory being true. This would give a ground framework for how free will should exist in this world, and just this notion could help the lives of countless people. This line of thought would support the current legal system (Zeki et al., 2004) without the need to ditch a physiologic concept of free will, as some have the habit of doing. They back a judicial system that is based on what is needed to maintain orderliness in society, without any regard to moral concepts and the need for an underlying understanding of free will. No, with this theory, we can align neurophysiology with the current judicial system. Nevertheless, this theory can also open the road for a new perspective in the approach to some diseases and conditions. Conditions like Schizophrenia it is known they harbor spontaneous activation of some regions of their brains, like those implicated in their auditory hallucinations (Hirano et al., 2015; Rolls, 2021). Other mental diseases could also benefit from this breakthrough; we could even make novel treatments that could target the CWF in these types of neurons. We could even understand how anesthetics work (Mashour, 2024), and we could lay down a framework of how consciousness operates inside our brains. And I think that this is a valuable question worthy of our attention.

## Data Availability

The original contributions presented in the study are included in the article/supplementary material, further inquiries can be directed to the corresponding author.

## Appendix

**Table A1:** Parameters and variables.

Symbol	Description
α, β	Amplitudes of Neuron States
g	Coupling Constant (soul-brain interaction)
λ	Soul’s Intentional Influence
$V_{i}$	Membrane Potential of Neuron i
ΔS	Change in Entropy
